# Mutation analysis of the *CHK2* gene in families with hereditary breast cancer

**DOI:** 10.1054/bjoc.2001.1858

**Published:** 2001-07

**Authors:** M Allinen, P Huusko, S Mäntyniemi, V Launonen, R Winqvist

**Affiliations:** Department of Clinical Genetics, University of Oulu/Oulu University Hospital, Oulu, Finland; Department of Mathematical Sciences, University of Oulu, Oulu, Finland; Department of Medical Genetics, Biomedicum Helsinki, University of Helsinki, Helsinki, Finland

**Keywords:** hereditary breast cancer, *CHK2* mutations, Li–Fraumeni-like syndrome

## Abstract

Recently CHK2 was functionally linked to the p53 pathway, and mutations in these two genes seem to result in a similar Li–Fraumeni syndrome (LFS) or Li–Fraumeni-like syndrome (LFL) multi-cancer phenotype frequently including breast cancer. As CHK2 has been found to bind and regulate BRCA1, the product of one of the 2 known major susceptibility genes to hereditary breast cancer, it also more directly makes *CHK2* a suitable candidate gene for hereditary predisposition to breast cancer. Here we have screened 79 Finnish hereditary breast cancer families for germline *CHK2* alterations. Twenty-one of these families also fulfilled the criteria for LFL or LFS. All families had previously been found negative for germline *BRCA1* *BRCA2* and *TP53* mutations, together explaining about 23% of hereditary predisposition to breast cancer in our country. Only one missense-type mutation, Ile^157^→Thr^157^, was detected. The high Ile^157^ → Thr^157^mutation frequency (6.5%) observed in healthy controls and the lack of other mutations suggest that *CHK2* does not contribute significantly to the hereditary breast cancer or LFL-associated breast cancer risk, at least not in the Finnish population. For Ile^157^ → Thr^157^our result deviates from what has been reported previously. © 2001 Cancer Research Campaign http://www.bjcancer.com


British Journal of Cancer (2001) 85(2), 209–212
© 2001 Cancer Research Campaign
doi: 10.1054/ bjoc.2001.1858, available online at http://www.idealibrary.com on IDEAL®

### Mutation analysis of the *CHK2* gene in families with hereditary breast cancer

**M Allinen[1], P Huusko[1], S Mäntyniemi[2], V Launonen[3] and R Winqvist[1]**

[1]Department of Clinical Genetics, University of Oulu/Oulu University Hospital, Oulu, Finland; [2]Department of Mathematical Sciences, University of Oulu, Oulu, Finland; [3]Department of Medical Genetics, Biomedicum Helsinki, University of Helsinki, Helsinki, Finland

**Summary** Recently CHK2 was functionally linked to the p53 pathway, and mutations in these two genes seem to result in a similar Li–Fraumeni syndrome (LFS) or Li–Fraumeni-like syndrome (LFL) multi-cancer phenotype frequently including breast cancer. As CHK2 has been found to bind and regulate BRCA1, the product of one of the 2 known major susceptibility genes to hereditary breast cancer, it also more directly makes *CHK2* a suitable candidate gene for hereditary predisposition to breast cancer. Here we have screened 79 Finnish hereditary breast cancer families for germline *CHK2* alterations. Twenty-one of these families also fulfilled the criteria for LFL or LFS. All families had previously been found negative for germline *BRCA1*, *BRCA2* and *TP53* mutations, together explaining about 23% of hereditary predisposition to breast cancer in our country. Only one missense-type mutation, $Ile^{157} \rightarrow Thr^{157}$, was detected. The high $Ile^{157} \rightarrow Thr^{157}$ mutation frequency (6.5%) observed in healthy controls and the lack of other mutations suggest that *CHK2* does not contribute significantly to the hereditary breast cancer or LFL-associated breast cancer risk, at least not in the Finnish population. For $Ile^{157} \rightarrow Thr^{157}$ our result deviates from what has been reported previously. © 2001 Cancer Research Campaign http://www.bjcancer.com

**Keywords**: hereditary breast cancer; *CHK2* mutations; Li–Fraumeni-like syndrome

It has been proposed that the known susceptibility genes account only for approximately 20–25% of the hereditary risk of getting breast cancer (Lichtenstein et al, 2000). Mutations in the 2 major breast cancer susceptibility genes, *BRCA1* and *BRCA2* (Miki et al, 1994; Wooster et al, 1995), have been found in only about 20% of Finnish high-risk breast cancer families (Vehmanen et al, 1997a, b; Huusko et al, 1998). Mutations in a third gene, *TP53*, appear to be responsible for a minor additional fraction of predisposition to breast cancer (reviewed in Easton, 1999). Recently, we studied the contribution of *TP53* mutations for breast cancer predisposition in Finland (Huusko et al, 1999; Rapakko et al, 2001). Mutations were found in only 3/108 (2.8%) of *BRCA1* and *BRCA2* mutation-negative families. In our studies, *TP53* changes occurred exclusively in those breast cancer families also displaying a Li–Fraumeni syndrome (LFS) or Li–Fraumeni-like syndrome (LFL) cancer background (e.g. sarcomas, breast cancer, leukaemia, and tumours of the central nervous system and adrenal cortex; Garber et al, 1990), with at least one case of bilateral disease. These observations clearly indicate that other breast cancer susceptibility genes must also be involved (Easton, 1999). Recently, a new susceptibility locus was identified in chromosome region 13q21 (Kainu et al, 2000). However, it has been estimated that this gene at the most would explain about 25% of the remaining *BRCA1/BRCA2* negative families (originating preferentially from the central and southern parts of the country), and that there still are additional breast cancer genes to be identified.

Bell et al (1999) identified germline *CHK2* mutations in *TP53*-negative LFS and LFL families. They suggested that *CHK2*, which encodes a protein kinase required for DNA damage and replication checkpoints, is another tumour suppressor gene along with *TP53* conferring predisposition to sarcoma, breast cancer and brain tumours. After DNA damage, ATM-dependent activation of both p53 and CHK2 occurs (reviewed in Prives and Hall, 1999). As CHK2 is capable of phosphorylating p53 at $Ser^{20}$ (Hirao et al, 2000), it appears to function as an intermediate kinase and thus plays a key role in connecting p53 to the response to double-stranded DNA breaks. Furthermore, CHK2 also binds to and regulates BRCA1 (Lee et al, 2000), and the phosphorylation of BRCA1 at $Ser^{988}$ is required for the release from CHK2. Wang et al (2000) suggested that BRCA1 could act as a scaffold protein that organizes different types of DNA damage sensors and then serves as an effector in response to DNA damage to coordinate repair.

Both the association to LFS/LFL and the regulatory control of BRCA1, encoded by one of the 2 known major susceptibility genes to hereditary breast cancer, makes *CHK2* a good candidate gene to search for involvement in the remaining unexplained cases of genetic predisposition to this disease. The search for *CHK2* mutations was performed on 79 Finnish families with indications of hereditary breast cancer, in which *BRCA1*, *BRCA2* and *TP53* mutations were previously excluded (Huusko et al, 1998, 1999; Rapakko et al, 2001). The validation of observed sequence alterations was done on cohorts of suitable cancer-free and unselected breast cancer individuals.

*Received 11 December 2000*
*Revised 6 March 2001*
*Accepted 20 March 2001*

*Correspondence to:* R Winqvist

### MATERIALS AND METHODS

The search for *CHK2* germline mutations included all exons and splice-site boundary regions and was performed on 79 families with hereditary breast cancer (Table 1) originating from the Oulu University Hospital area. From some of the cancer families multiple affected individuals were studied. In addition, from 3 of

**Table 1** Summary of the classification of the studied families[a]

| Phenotype | Number of families |
|---|---|
| All studied breast cancer families | 79 |
| Families with implications of hereditary breast cancer only | 58 |
| Breast cancer families also fulfilling the LFL criteria | 20 |
| Breast cancer families also fulfilling the LFS criteria | 1 |

[a]For the inclusion criteria for each category, see the Materials and Methods section.

the families unaffected members were also analysed for a specific gene alteration. Of the total of 98 breast cancer cases, 7 (7%) were identified at or below age 35, 23 (24%) between ages 36–45, 49 (50%) between ages 46–60, and 19 (19%) at or above age 61. Fifty-eight families met the criteria for moderate- to high-risk hereditary breast cancer only, 20 families for both hereditary breast cancer and LFL, and one family for both hereditary breast cancer and LFS. The used criteria for hereditary breast cancer were one or more of the following: (1) at least 3 (2 in combination with other selection criteria) cases of breast cancer in first- or second-degree relatives; (2) early disease onset (≤35 years alone, or <45 in combination with other inclusion criteria); (3) bilateral breast cancer; or (4) multiple tumours including breast cancer in the same individual. The criteria for LFL/LFS were as in Birch et al (1994) and Eng et al (1997). Informed consent to obtain pedigree data and blood specimen for a study on cancer susceptibility gene mutations was obtained from all patients. Control DNA samples from blood were derived from 200 anonymous cancer-free donors and 259 unselected breast cancer patients. Approval to perform the study was obtained from the Ethical Board of the Northern Ostrobotnia Health Care District and the Finnish Ministry of Social Affairs and Health.

DNA extraction from blood lymphocyte specimens was performed using the standard phenol-chloroform method. The screening for *CHK2* mutations was done by conformation-sensitive gel electrophoresis (CSGE) analysis (Huusko et al, 1998). Samples with a band-shift were reamplified and purified with the QIAquick PCR purification Kit (Qiagen). Sequencing analysis was performed with the Li-Cor IR$^2$ 4200-S DNA Analysis system (Li-Cor Inc, Lincoln, USA) and using the SequiTherm EXCEL™II DNA Sequencing Kit-LC (Epicentre Technologies), following the protocol provided by Li-Cor. Oligonucleotides for CSGE analysis were synthesized based on available *CHK2* genomic sequences (Genbank accession number AL117330). Additional oligos for CSGE and sequencing were designed by using the Primer3 software. Primer sequences and PCR conditions for CSGE and sequencing are available upon request.

Mutation frequency differencies between the tested groups were analysed in Bayesian framework (Gelman et al, 1995). Unlike the Chi-square test, this approach provides the probabilities for the presented hypothesis being both true and false. Furthermore, in the Bayesian model none of the expected values are fixed, which results in a more plausible statistical estimate. The probability model was set up assuming that the number of mutations follow poisson distribution with mean $\lambda_i = \theta_i N_i$, when the number of individuals is $N_i$ and the mutation frequency is $\theta_i$. Also, $\theta_i$ was assumed to follow Beta (1, 1) = Unif (0, 1) distribution. Formally:

xi|Ni,θi ~ Poisson (θiNi)
θi ~ Beta(1,1)

The comparisons between mutation frequencies in different groups were performed by calculating the ratio of the frequencies, $R_{ij} = \theta_i/\theta_j$. Posterior distributions of the model parameters were obtained by Monte Carlo Markov Chain stimulation, which was carried out with WinBUGS 1.3 software. Also, for $H_0$ (estimating how well the frequency observed in one group equals that in the comparison group) traditional Chi-square test calculations were performed, using $P = 0.01$ as cut-off value for statistical significance.

### RESULTS AND DISCUSSION

In the current study, only one missense-type mutation, Ile$^{157}$ → Thr$^{157}$, was detected within the protein-encoding region of the *CHK2* gene. This alteration was the same as that previously reported by Bell et al (1999). In addition, 2 changes in intronic sequences were found. No splice-site alterations were observed.

Ile$^{157}$ → Thr$^{157}$ was seen in 7/79 (8.9%) of breast cancer families (group 1). Four of these 7 families also met the criteria for LFL. In 2 of the mutation-positive families, the mutation segregated ambiguously with the cancer phenotype (Figure 1). In family #5, a woman with breast cancer diagnosed at 80 carried the mutation, whereas her unaffected 47-year-old daughter did not. However, the proband's unaffected 63-year-old niece was found to be a mutation carrier. In family #7, a mother and daughter diagnosed with breast cancer at ages 64 and 49, respectively, were both mutation carriers, but the other daughter who had breast cancer at 40 was not. In addition, Ile$^{157}$ → Thr$^{157}$ was found in 13/200 (6.5%) of anonymous cancer-free blood donors (group 2), and 10/259 (3.9%) of unselected breast cancer cases (group 3).

Using the Bayesian model, none of the probabilities for the mutation frequencies being higher among hereditary breast cancer patients reached 0.99, the minimum value to prove that the observed incidence is higher than expected. The obtained probabilities were 0.78 (group 1 vs 2), 0.11 (group 2 vs 3) and 0.96 (group 1 vs 3). To estimate how well the frequency observed in one group equals that in a comparison group, traditional Chi-square test calculations were made. The obtained values were 0.72 ($P = 0.395$), 2.96 ($P = 0.085$) and 5.53 ($P = 0.019$), respectively, and thus statistically insignificant.

As implied by the performed statistical analysis, our observation for group 2 is in contrast to the previous finding of Bell et al (1999), who did not detect the Ile$^{157}$ → Thr$^{157}$ missense mutation among any of the 50 healthy individuals used as controls, but only in one LFL individual with 3 primary tumours (breast, melanoma and lung) and no other reported family history of cancer. Although Ile$^{157}$ → Thr$^{157}$ is located within the forkhead-associated (FHA) domain, which is a highly conserved 60-amino acid protein-interaction domain essential for activation of the CHK2 yeast homolog Rad53 in response to DNA damage (Sun et al, 1998), the

© 2001 Cancer Research Campaign

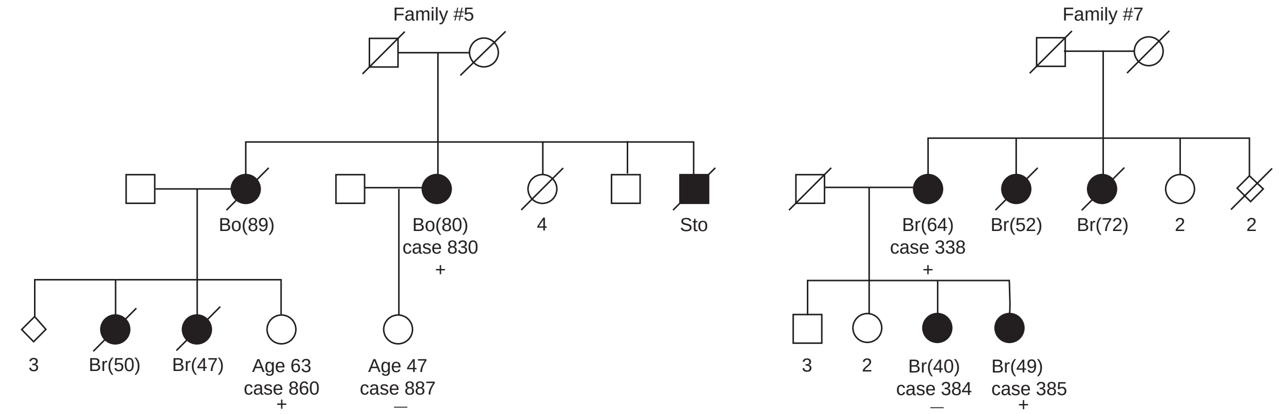

**Figure 1** The pedigrees of two *CHK2* Ile$^{157}$ → Thr$^{157}$ positive Finnish breast cancer families showing ambiguous allele segregation. Tumours: Br, breast; Bo, bone; Sto, stomach. The age at diagnosis, when known, is marked after the malignancy. (+) = mutation carrier, (–) = not a carrier. The case numbers of the individuals analysed are shown above the carrier status high mutation frequency (6.5%) now observed in healthy Finnish controls suggests that Ile$^{157}$ → Thr$^{157}$ is not, at least alone, a mutation resulting in predisposition to cancer. The statistical analysis also shows that Ile$^{157}$ → Thr$^{157}$ is not significantly enriched among breast cancer patients having hereditary disease background (including LFL). Furthermore, the ambiguous segregation in the studied informative cancer families suggests that this alteration is rather a polymorphism than a deleterious mutation. This notion is also supported by the recent observation of Wu et al (2000), who found that CHK2 protein carrying the Ile$^{157}$ → Thr$^{157}$ change has similar kinase activity, expression levels and subcellular localization as endogenous CHK2. Also, like wild-type CHK2, the mutant protein is activated following gamma radiation. However, it is still unclear whether Ile$^{157}$ → Thr$^{157}$ has other effects on cellular phenotype, or possibly acts as a genetic modifier on a breast cancer predisposing background.

Bell and coworkers (1999) screened 4 LFS and 18 LFL cases, and detected *CHK2* mutations in 3 of the studied families (13.6%). Therefore, a similar incidence of *CHK2* mutations was initially expected also among the 21 LFL and LFS families studied by us. Together with the recent results of Sodha et al (2000) it now appears that only 1 of 3 *CHK2* mutations originally reported by Bell et al (1999) is a true disease-causing change, and thus the expected frequency of *CHK2* mutations in LFS and LFL families would be lower than was initially assumed.

Due to the duplications of the 3′ genomic sequences of *CHK2* reported by Sodha et al (2000), atypical banding in CSGE was observed while analysing the terminal exons 10–14, encoding most of the protein kinase domain (data not shown). CSGE analysis is based on homo-and heteroduplex formation between wild-type and mutated alleles, leading to altered mobility of different types of DNA duplexes on a denaturating polyacrylamide gel. Körkkö et al (1998) showed that it is possible to detect more than one kind of mismatch in the same PCR product, by the appearance of new heteroduplex bands in CSGE. Therefore, instead of a single band (e.g. homoduplex) indicating the lack of mutation, genomic loci coamplified in PCR with a tested segment of *CHK2* exon 10–14 would in CSGE analysis result in additional bands (e.g. one or more heteroduplexes). For that reason, we concluded that screening for samples displaying a different banding pattern in CSGE could at least provide a rough idea whether the analysed exons contain alterations or not. The banding patterns for exons 10–14 in our study, however, were similar in all screened DNA samples (data not shown). To conclusively exclude the presence of mutations in exons 10–14, this negative result should be confirmed by using allele-specific PCR amplification. Unfortunately, in the current study fresh sample material or breast cancer cell lines to perform this type of analysis were not available.

As no other mutations besides Ile$^{157}$ → Thr$^{157}$ were detected within the protein-encoding region of the *CHK2* gene, our results suggest that *CHK2* does not play a significant role as predisposing factor for hereditary breast cancer, or LFL showing excessive cases of breast cancer, at least in the Finnish population. Larger studies will be needed to more carefully evaluate the significance of *CHK2* alterations in predisposition to cancers related to LFS, as well as to estimate the possible effects of founder mutations in different populations.

### ACKNOWLEDGEMENTS

We thank MD PhDs Guillermo Blanco, Jaakko Leisti and Ulla Puistola for help in patient contacts, PhD Åke Borg for discussions, PhD Heli Nevanlinna for help with the PCR oligonucleotides, PhD Pertti Sistonen at the Finnish Red Cross Blood Transfusion Service in Helsinki for providing the blood samples from anonymous cancer-free blood donors, and Ms Marika Kujala for technical assistance. The support from the Academy of Finland, the Finnish Cancer Society, the Cancer Foundation of Northern Finland, the University of Oulu, the Oulu University Hospital, and the Finnish Breast Cancer Group is gratefully acknowledged. We also thank all patients for volunteering to participate in this study.

### REFERENCES

Bell DW, Varley JM, Szydlo TE, Kang DH, Wahrer DCR, Shannon KE, Lubratovich M, Verselis SJ, Isselbacher KJ, Fraumeni JF, Birch JM, Li FP, Garber JE and Haber DA (1999) Heterozygous germ line *hCHK2* mutations in Li-Fraumeni syndrome. *Science* **286**: 2528–2531

Birch JM, Hartley AL, Tricker KJ, Prosser J, Condie A, Kelsey AM, Harris M, Morris Jones PH, Binchy A, Crowther D, Craft AW, Eden OB, Evans GR, Thompson E, Mann JR, Martin J, Mitchell ELD and Santibanez-Koref MF

(1994) Prevalence and diversity of constitutional mutations in the *p53* gene among 21 Li-Fraumeni families. *Cancer Res* **54**: 1298–1304

Easton DF (1999) How many more breast cancer predisposition genes are there? *Br Cancer Res* **1**: 14–17

Eng C, Schneider K, Fraumeni JF and Li FP (1997) Third international workshop on collaborative interdisciplinary studies of *p53* and other predisposing genes in Li-Fraumeni syndrome. *Cancer Epidemiol Biomark Prev* **6**: 379–383

Garber JE, Goldstein AM, Kantor AF, Dreyfus MG, Fraumeni JF and Li FP (1991) Follow-up study of twenty-four families with Li-Fraumeni syndrome. *Cancer Res* **51**: 6094–6097

Gelman A, Carlin JB, Stern HS and Rubin DB (1995) Bayesian Data Analysis. Chapman & Hall, London, pp. 3–25, 48–51

GenBank, http://www.ncbi.nlm.nih.gov/Genbank/index.html (for nucleotide sequence [accession number AL117330])

Hirao A, Kong Y-Y, Matsuoka S, Wakeham A, Ruland J, Yoshida H, Liu D, Elledge SJ and Mak TW (2000) DNA damage-induced activation of p53 by the checkpoint kinase Chk2. *Science* **287**: 1824–1827

Huusko P, Pääkkönen K, Launonen V, Pöyhönen M, Blanco G, Kauppila A, Puistola U, Kiviniemi H, Kujala M, Leisti J and Winqvist R (1998) Evidence of founder mutations in Finnish *BRCA1* and *BRCA2* families. *Am J Hum Genet* **62**: 1544–1548

Huusko P, Castrén K, Launonen V, Soini Y, Pääkkönen K, Leisti J, Vähäkangas K and Winqvist R (1999) Germ-line TP53 mutations in Finnish cancer families exhibiting features of the Li-Fraumeni syndrome and negative for *BRCA1* and *BRCA2*. *Cancer Genet Cytogenet* **112**: 9–14

Kainu T, Juo SH, Desper R, Schaffer AA, Gillanders E, Rozenblum E, Freas-Lutz D, Weaver D, Stephan D, Bailey-Wilson J, Kallioniemi O-P, Tirkkonen M, Syrjäkoski K, Kuukasjärvi T, Koivisto P, Karhu R, Holli K, Arason A, Johannesdottir G, Bergthorsson JT, Johannsdottir H, Egilsson V, Björk Barkadottir R, Johansson O, Haraldsson K, Sandberg T, Holmberg E, Grönberg H, Olsson H, Borg Å, Vehmanen P, Eerola H, Heikkilä P, Pyrhönen S and Nevanlinna H (2000) Somatic deletions in hereditary breast cancers implicate 13q21 as a putative novel breast cancer susceptibility locus. *Proc Natl Acad Sci USA* **97**: 9603–9608

Körkkö J, Annunen S, Pihlajamaa T, Prockop DJ and Ala-Kokko L (1998) Conformation sensitive gel electrophoresis for simple and accurate detection of mutations: Comparison with denaturing gradient gel electrophoresis and nucleotide sequencing. *Proc Natl Acad Sci USA* **95**: 1681–1685

Lee J-S, Collins KM, Brown AL, Lee C-H and Chung JH (2000) hCdsl-mediated phosphorylation of BRCA1 regulates the DNA damage response. *Nature* **404**: 201–204

Lichtenstein P, Holm NV, Verkasalo PK, Iliadou A, Kaprio J, Koskenvuo M, Pukkala E, Skytthe A and Hemminki K (2000) Environmental and heritable factors in the causation of cancer. *N Engl J Med* **343**: 78–85

Miki Y, Swensen J, Shattuck-Eidens D, Futreal PD, Harshman K, Tavtigian S, Liu Q, Cochran C, Bennett LM, Ding W, Bell R, Rosenthal J, Hussey C, Tran T, McClure M, Frye C, Hattier T, Phelps R, Haugen-Strano A, Katcher H, Yakumo K, Gholami Z, Shaffer D, Stone S, Bayer S, Wray C, Bogden R, Dayananth P, Ward J, Tonin P, Narod S, Bristow BK, Norris FH, Helvering L, Morrison P, Rosteck P, Lai M, Barrett JC, Lewis C, Neuhausen S, Cannon-Albright L, Goldgar D, Wiseman R, Kamb A and Skolnick MH (1994) A strong candidate for the breast and ovarian cancer susceptibility gene *BRCA1*. *Science* **266**: 66–71

Primer3 software, (http://www-genome.wi.mit.edu/cgibin/primer/primer3_www.cgi)

Prives C and Hall PA (1999) The p53 pathway. *J Pathol* **187**: 112–126

Rapakko K*, Allinen M*, Syrjäkoski K, Vahteristo P, Huusko P, Vähäkangas K, Eerola H, Kainu T, Kallioniemi, Nevanlinna H and Winqvist R *Equal contribution (2001) Germline *TP53* alterations in Finnish breast cancer families are rare and occur at conserved mutation-prone sites. *Br J Cancer* **84**: 116–119

Sodha N, Williams R, Mangion J, Bullock SL, Yuille MR and Eeles RA (2000) Screening *hCHK2* for mutations. **289**: 359a

Sun Z, Hsiao J, Fay DS and Stern DF (1998) Rad53 FHA domain associated with phosphorylated Rad9 in the DNA damage checkpoint. *Science* **281**: 272–274

Vehmanen P, Friedman LS, Eerola H, McClure M, Ward B, Sarantaus L, Kainu T, Syrjäkoski K, Pyrhönen S, Kallioniemi O-P, Muhonen T, Luce M, Frank TS and Nevanlinna H (1997*a*) Low porpotion of *BRCA1* and *BRCA2* mutations in Finnish breast cancer families: evidence for additional susceptibility genes. *Hum Mol Genet* **6**: 2309–2315

Vehmanen P, Friedman LS, Eerola H, Sarantaus L, Pyrhönen S, Ponder ABJ, Muhonen T and Nevanlinna H (1997*b*) A low porpotion of *BRCA2* mutations in Finnish breast cancer families. *Am J Hum Genet* **60**: 1050–1058

Wang Y, Cortez D, Yazdi P, Neff N, Elledge SJ and Qin J (2000) BASC, a super complex of BRCA1-associated proteins involved in the recognition and repair of aberrant DNA structures. *Genes Dev* **14**: 927–939

Wooster R, Bignell G, Lancaster J, Swift S, Seal S, Mangion J, Collins N, Gregory S, Gumbs C, Micklem G, Barfoot R, Hamoudi R, Patel S, Rice C, Biggs P, Hashim Y, Smith A, Connor F, Arason A, Gudmundsson J, Ficenec D, Kelsell D, Ford D, Tonin P, Bishop DT, Spurr NK, Ponder BAJ, Eeles R, Peto J, Devilee P, Cornelisse C, Lynch H, Narod S, Lenoir G, Egilsson V, Barkardottir RB, Easton DF, Bentley DR, Futreal PA, Ashworth A and Stratton MR (1995) Indentification of the breast cancer susceptibility gene *BRCA2*. *Nature* **378**: 789–792

Wu X, Webster SR and Chen J (2001) Characterization of tumor-associated *Chk2* mutations. *J Biol Chem* **276**: 2971–2974

*© 2001 Cancer Research Campaign*
